# Maternal High-Fat Diet Leads to Non-alcoholic Fatty Liver Disease Through Upregulating Hepatic *SCD1* Expression in Neonate Rats

**DOI:** 10.3389/fnut.2020.581723

**Published:** 2020-11-17

**Authors:** Baige Cao, Chongxiao Liu, Qianren Zhang, Yan Dong

**Affiliations:** ^1^Department of Endocrinology, Xinhua Hospital, Shanghai Jiao Tong University School of Medicine, Shanghai, China; ^2^Shanghai Institute for Pediatric Research, Shanghai Jiao Tong University School of Medicine, Shanghai, China; ^3^Department of Endocrinology, Seventh People's Hospital of Shanghai University of Traditional Chinese Medicine, Shanghai, China

**Keywords:** maternal high fat diet, inflammation, lipid metabolism, SCD1 expression, HepG2 cells

## Abstract

Non-alcoholic fatty liver disease (NAFLD) has become the leading cause of liver disease in children, with evidence that the maternal diet and the early life nutritional environment are potential risk for such disease. This study was aimed to investigate the effects of maternal high-fat diet (HFD) on the occurrence of NAFLD in offspring rats and the underlying mechanisms. In this study, the incidence of NAFLD was compared in F1 offspring rats between the maternal HFD group and standard chow (SC) group. In addition, the expression levels of inflammatory cytokines in the placenta, in the umbilical cord blood, and in the livers of neonate offsprings were compared between two groups. HepG2 cells were treated with recombinant IL6 (rIL6) to assess stearoyl-CoA desaturase 1 (SCD1) expression and lipid synthesis in an inflammatory condition. Lipid accumulation was assayed in both *SCD1* overexpression and interference HepG2 cells as well as in neonatal rats. Our results showed that HFD exposure before and throughout the pregnancy induced the elevated hepatic TG content of F1 neonates. The levels of inflammatory cytokines in the placenta, umbilical cord blood, and the livers of HFD F1 neonates were significantly higher than those of the SC group. In addition, rIL6 treatment led to TG accumulation accompanied by the upregulation of *SCD1* in HepG2 cell lines. Overexpression of *SCD1* led to the accumulation of TG contents in HepG2 cells, whereas *Scd1* knockdown attenuated the effects of rIL6 treatment. Overexpression of *SCD1* in F1 neonatal rats led to hepatic lipid accumulation. Our study indicated that maternal HFD led to intrauterine inflammation, which subsequently caused transgenerationally abnormal hepatic lipid metabolism of F1 neonates. This modulation might be mediated by upregulating *SCD1* expression in hepatic cells.

## Introduction

Non-alcoholic fatty liver disease (NAFLD) is a series of hepatic abnormalities including simple hepatic steatosis and non-alcoholic steatohepatitis (NASH) with hepatic fibrosis and chronic inflammation. NAFLD has become a global health concern. It is the second most common indication for liver transplantation in adults (1), adding to the existing global health care burden in the developed world. Obesity is among the major risk factors for the occurrence of NAFLD. Of note, recent investigations reveal that the pathogenesis of NAFLD is comprehensive and multifactorial (2, 3), among which suboptimal maternal nutrition is widely implicated. Two major damages led to the occurrence of NAFLD, which includes lipid accumulation in the hepatocytes and inflammation injury. Previous studies showed that IL6 was associated with insulin resistance and enhanced obesity and liver steatosis in HFD-fed mice (4). Similarly, our previous results showed that maternal high-fat diet (HFD) during pregnancy resulted in the occurrence of NAFLD in offsprings at weaning.

Inappropriate intrauterine nutrition can in part drive the abnormal metabolic processes, making the fetuses, neonates, and infants more susceptible to metabolic disorders during their developmental process, such as fatty liver disease, insulin resistance, and cardiovascular disease (5–7). For instance, obesity or gestational diabetes is among the major risk factors for the occurrence of clinical symptoms in the offsprings (8). Maternal HFD induces self-obesity and subsequent inflammatory responses, leading to compromised vascular structure, blood flow, and fetal oxygen supply in the placenta of maternal obesity models (9, 10). The placenta is the maternal–fetal interface responsible for nutrient supply to the fetus. Pregnant women with obesity or gestational diabetes mellitus (GDM) with imbalance in intrauterine nutrition (11, 12) have increased incidence of placenta ischemia, hypoxia, high cytokine production, and oxidative stress (9, 13–15). This will in turn result in abnormal lipogenesis and inflammation (16, 17) and in the upregulation of fatty acid synthesis genes and downregulation of β-oxidation-related gene expression in offspring livers (18).

Our previous study in a rat HFD model revealed that maternal HFD during pregnancy and lactation resulted in weight gain, impaired glucose tolerance, and the occurrence of NAFLD in the offsprings at 3 weeks of weaning (19). Interestingly, the body weight of newborn rats was lower than that of control rats. Transcriptome analysis of the livers from 3-week-old offsprings (F1) showed that a panel of lipid metabolism-related genes was upregulated in F1-HFD rats, among which stearoyl-CoA desaturase 1 (*Scd1*) was 25 times higher than that from the F1-CON group. *SCD1* is mainly responsible for the conversion of saturated fatty acids (SFAs) into monounsaturated fatty acids (MUFAs) in the liver. It has been shown previously that diet regulates hepatic *SCD1* expression. For instance, short-term high-carbohydrate diet can upregulate the activity of SCD1 and activate the *de novo* synthesis of triglycerides (TGs) (20). Lorena et al. also confirmed that HFD caused the inflammatory state in the rats with the upregulation of *Scd1* (21). Considering the critical role of SCD1 in lipid metabolism, it could be hypothesized that maternal HFD might be one of the triggering factors related to upregulation of *SCD1* and the subsequent metabolic disorders in their offsprings, through regulating the balance of intrauterine nutrition and inflammation (22). The underlying mechanism remains unclear and needs further investigation.

In order to explore the effect of maternal HFD in pathogenesis of neonatal NAFLD and its underlying mechanisms, we conducted the study both *in vivo* and *in vitro* by using an HFD rat model and HepG2 cells. Intrauterine inflammation and offspring hepatic *Scd1* gene expression were assessed to determine the direct effects of HFD on the incidence of NAFLD in neonates *in vivo*. HepG2 cells were used to determine the mechanism of SCD1 in the regulation of lipid metabolism under inflammatory conditions. Additionally, we also explored the role of IL6 in the process of neonatal NAFLD caused by maternal HFD.

## Materials and Methods

### Animals and Treatment

Female Sprague–Dawley (SD) rats (3-week-old) were purchased from the Shanghai Experimental Animal Center of the Chinese Academy of Sciences (Shanghai, China). All rats were housed routinely in the animal facility of Xinhua Hospital affiliated to Shanghai Jiao Tong University School of Medicine and were approved by the University Animal Use Committee. All mice (*n* = 12) were randomly subgrouped into a control group with free access to the standard chow diet (12.9% fat, 25.5% protein, and 61.6% carbohydrate) (F0-CON) (*n* = 6) or HFD group (45.0% fat, 20.0% protein, and 35.0% carbohydrate) for 8 weeks (F0-HFD) (*n* = 6). Eight weeks later, female rats were mated with age-matched male SD rats. All female rats were maintained on their diets throughout the pregnancy. In the *in vivo Scd1* intervention experiment, neonates from six different F0-CON pregnant rats were divided into three groups: F1-blank group (*n* = 6) without the injection, F1-rAAV-NC group (*n* = 6) with the infection of the corresponding control vector, and F1-rAAV-Scd1 group (*n* = 6) with the injection of rAAV-Scd1 overexpression vector (Han Heng, Shanghai, China) through the tail vein. All neonates were fed by F0-CON rats. The male-to-female ratio in each group is 1:1. Litter size was adjusted to standardize nutrition until weaning.

### Sample Collection

After full-term pregnancy, female rats were anesthetized by intraperitoneal injection of 1% pentobarbital sodium 40 mg/kg. The livers from the neonates, female rats, and maternal placenta were resected and frozen in liquid nitrogen for further analysis. Umbilical cord blood was collected for the measurement of inflammatory cytokines. At 3 weeks old, the offspring rats were anesthetized, and liver tissues were resected and frozen in liquid nitrogen for further analysis.

### Determination of Lipid Metabolites

After accurate weighing, the lysate was added at a rate of 20 μl per 1 mg of tissue. The tissue was ground with a 2 ml homogenizer and set aside for 15 min. TG and cholesterol levels in the liver tissues were determined by TG assay kit and cholesterol assay kit (both from Applygen Company, Beijing, China), respectively, according to the manufacturer's instructions.

### Liver Histology

Fresh liver tissues (three sections were examined for each rat) were fixed in 10% paraformaldehyde solution and paraffin embedded prior to sectioning. All sections were stained with hematoxylin and eosin (H&E) and assessed for steatosis. The samples embedded in an optimal-cutting-temperature compound were stained with freshly prepared oil red stock solution. The nuclei of liver cells were counterstained in hematoxylin in order to verify lipid droplet. The sections were observed under a Leica DMI3000B microscope (Leica, Solms, Germany).

### Glucose Tolerance Test (GTT)

Intraperitoneal glucose tolerance tests (IPGTTs) were performed after 16 h of fasting in dams. Animals were intraperitoneally (i.p.) injected with the glucose at 2 mg/g of body weight. Blood was taken from tail blood at 0, 30, 60, and 120 min after the injection, and the glucose levels were analyzed by using the OneTouch Ultra system. The area under the curve (AUC) of IPGTTs between any two time points was calculated by the trapezoid approximation formula: [(Time difference in minutes between sequential reads) ^*^ (Glucose level 1st time point + Glucose level 2nd time point)/2] (23).

### Elisa

Umbilical cord blood was taken from 12 pregnant rats (gestation days 21–24), with a sterile syringe washed with 6% EDTA, and then centrifuged, and the supernatant was obtained to measure the levels of serum IL6, IL-1β, and TNF-α via the ELISA (Cell Biolabs, San Diego, CA) according to the manufacturer's instructions.

### Cell Culture

The HepG2 cell line was obtained from American Type Culture Collection (ATCC; Manassas, VA, United States) and cultured in Dulbecco's modified eagle medium (DMEM) containing 10% fetal bovine serum (FBS) (Gibco, CA, United States) at 37°C in the atmosphere of 5% CO_2_. In some experiments, recombinant IL6 (rIL6) (PeproTech, United States) (40 ng/ml) was added during the culture.

### Oil Red O Staining and Intracellular TG Assay

Oil red powder, 0.15 g (Sigma, United States), was dissolved in 30 ml isopropanol and stored in a dark place. The cells were washed twice with phosphate-buffered saline (PBS) and fixed with 4% paraformaldehyde for 30 min in the culture plates. Oil red stock solution was diluted with distilled water (ratio = 3:2) and added to the plate for 15 min. The plates were rinsed with 60% isopropanol and counterstained with hematoxylin for 10 s. After washing with distilled water, the cells in the plates were investigated under a Leica DMI3000B microscope (Leica, Germany).

The intracellular TG levels in HepG2 cells were determined by using a TG quantification kit (Applygen, Beijing, China) according to the manufacturer's instructions. Cellular TG levels were normalized to their protein concentrations.

### Small Interfering RNA (siRNA) and Plasmid Transfection

HepG2 cells were transfected with 50 nM Scd1-specific siRNA (GenePharma, Shanghai, China) or 2 ng SCD1 overexpression vector pEX-Scd1 and the corresponding control vector (GenePharma, Shanghai, China) by using Lipofectamine 2000 (Invitrogen, United States) according to the manufacturer's instructions in Opti-MEM medium (Gibco, CA, United States). The medium was replaced with DMEM with 10% FBS after the transfection for 6 h. The experiments were performed 24 h after the transfection.

### Quantitative Real-Time Polymerase Chain Reaction

Total RNA was extracted from hepatic tissues and HepG2 cells using TRIzol reagent (Invitrogen, USA). cDNA was prepared using the PrimeScript RT Reagent Kit (Takara, Shiga, Japan). Quantitative real-time polymerase chain reaction (Q-PCR) was performed by using a SYBR Premix Ex Taq kit (cat. no. RR420A; Takara, Japan) according to the manufacturer's instruction. Primers of target genes were listed in Supplementary Tables 1, 2. Relative abundance of target genes was calculated and normalized with housekeeping gene 18S rRNA represented by 2^−ΔΔCt^ values.

### Western Blot Analysis

The protein levels in maternal placenta and livers were determined by western blot analysis using anti-IL6, anti-TNF-α, anti-IL-1β, anti-PPARα, and anti-ACSL3 (all from ABclonal, Wuhan, China); anti-SCD1 (Abcam, Cambridge, MA); anti-SREBP1c (CST, Danvers, MA); and anti-FASN (CST, Danvers, MA) antibodies. Total proteins were extracted and subsequently separated by 8–12% SDS-PAGE gels. Proteins were transferred onto PVDF membranes and probed with corresponding antibodies overnight at 4°C, followed by incubation with anti-mouse or anti-rabbit secondary antibodies (Beyotime, Shanghai, China) for 1 h at room temperature. The proteins were visualized by using a Western chemiluminescent HRP substrate (Millipore Corporation, United States).

### Statistical Analysis

All data were presented as mean ± standard error of the mean (SEM). A two-tailed Student *t*-test was applied to compare the mean values between two groups, and differences between multiple groups were analyzed using a one-way analysis of variance test, followed by least significant difference analyses. Statistical analysis was performed by using SPSS v.20.0 (IBM Inc., Chicago, IL). A *P* < 0.05 was considered statistically significant.

## Results

### Maternal HFD Throughout Pregnancy Induces Liver Inflammation and *Scd1* Overexpression in Neonate Rats

**Figure 1:**
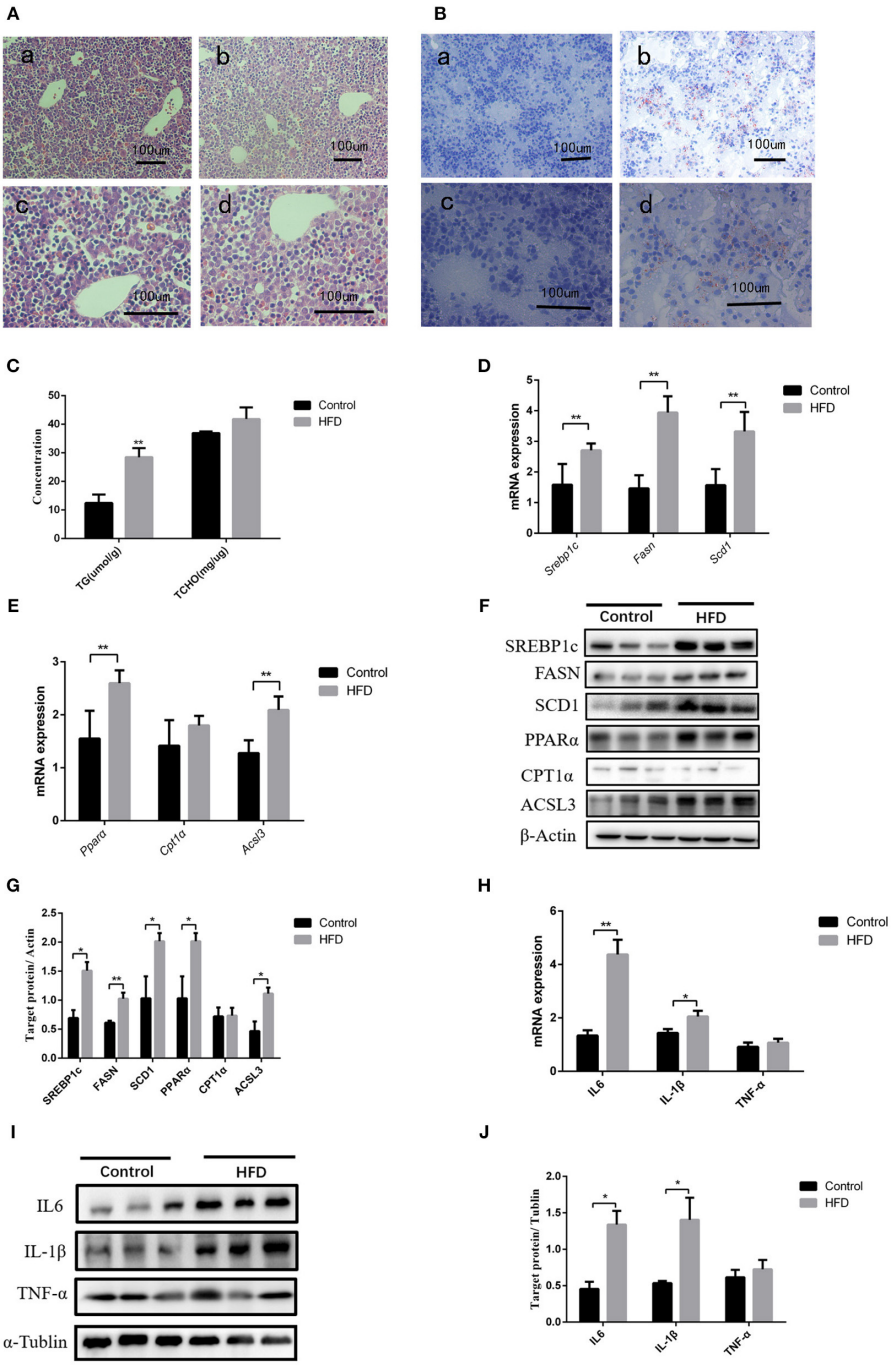
Pathological phenotypes and gene expression of lipid metabolism and inflammatory genes in neonate livers. **(A)** Hematoxylin and eosin (H&E) staining of neonate livers from F0-CON dams [(a): 200X; (b): 400X] and F0-HFD dams [(c): 200X; (d): 400X]. **(B)** Oil red O staining of neonate livers from F0-CON dams [(a): 200X; (b): 400X] and F0-HFD dams [(c): 200X; (d):400X]. **(C)** Comparison of hepatic lipid levels between the neonates from F0-CON and F0-HFD dams. **(D)** Comparison of gene expression levels related to lipid synthesis between the neonates from F0-CON and F0-HFD dams. **(E)** Comparison of gene expression levels related to lipid oxidation between the neonates from F0-CON and F0-HFD dams. **(F)** Protein levels in lipid metabolism between the neonates from F0-CON and F0-HFD dams. **(G)** Quantitative comparison of proteins in lipid metabolism between the neonates from F0-CON and F0-HFD dams. **(H)** Comparison of hepatic cytokine gene expression levels between the neonates from F0-CON and F0-HFD dams. **(I)** Protein levels of hepatic cytokine between the neonates from F0-CON and F0-HFD dams. **(J)** Quantitative comparison of hepatic cytokine protein levels between the neonates from F0-CON and F0-HFD dams. **P < 0.01; *P < 0.05.

After full-term pregnancy, mild ballooning degeneration and a large amount of lipid deposition were observed in liver cells of the dams fed with HFD compared to those fed normal diet during the pregnancy (Figures 1A,B). TG levels in neonate livers were increased when compared with those from normal-diet maternal rats (12.49 ± 2.95 vs. 28.46 ± 3.23 μmol/L, *P* < 0.01). However, the cholesterol content did not change significantly (Figure 1C). The Q-PCR results showed that the genes involved in lipid synthesis and β-oxidation, including *Srebp1c, Fasn, Scd1, Ppar*α, *Cpt1*α, and *Acsl3*, were upregulated (Figures 1D,E), which was confirmed by western blot results (Figures 1F,G). In addition, the expression of inflammatory cytokines including IL6 and IL-1β was increased in liver tissues of neonatal rats, whereas TNF-α level has no significant difference between control and HFD neonates (Figures 1H–J). Therefore, abnormal lipid metabolism might occur in the livers of the neonates from HFD maternal pregnancy.

### Maternal HFD Leads to Self-Obesity and Intrauterine Inflammation

**Figure 2:**
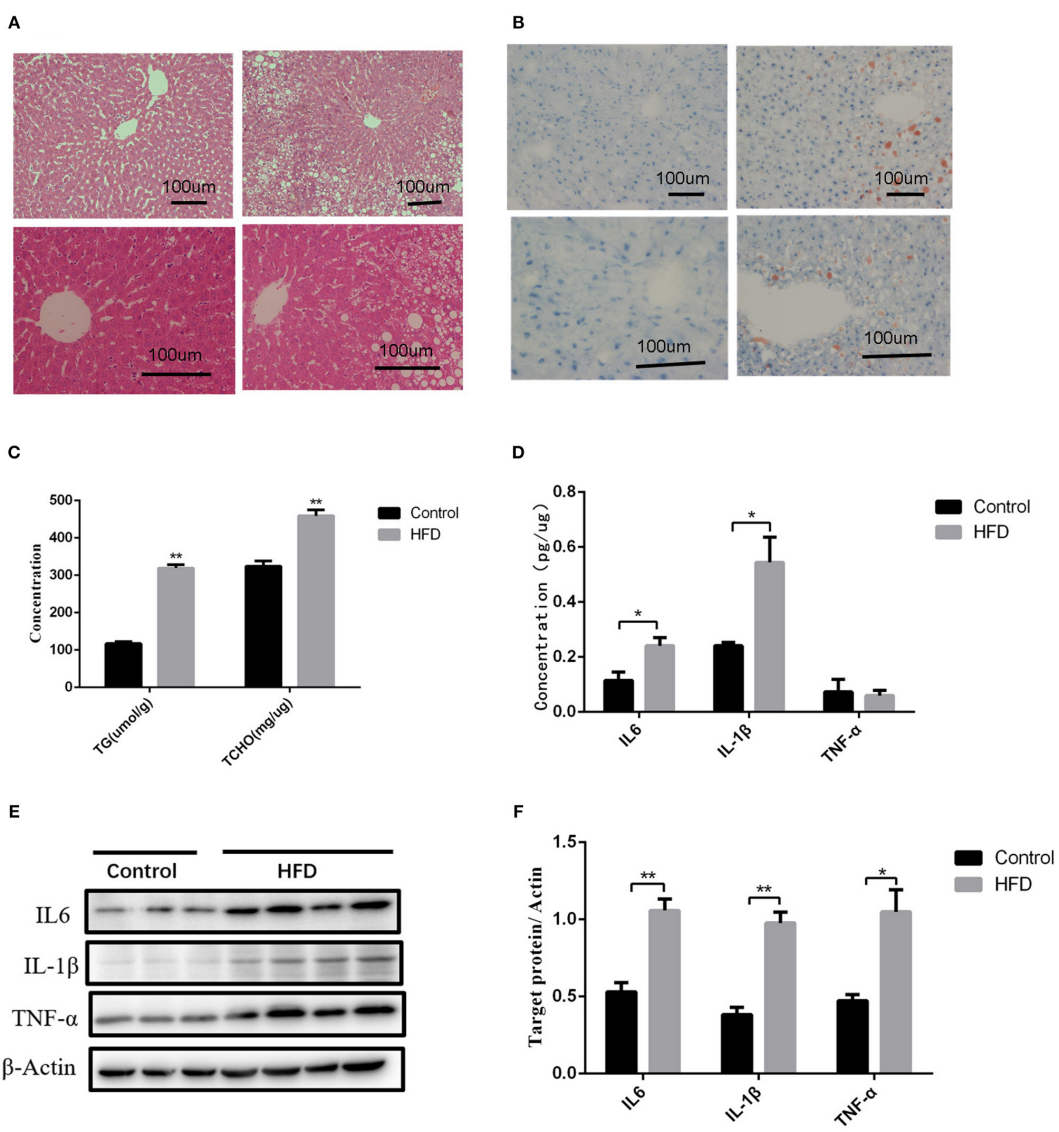
Liver pathology and inflammation status in the placental and umbilical cord in maternity dams. **(A)** Hematoxylin and eosin (H&E) staining of livers from F0-CON dams [(a): 200X; (b): 400X] and F0-HFD dams [(c): 200X; (d): 400X] after full-term pregnancy. **(B)** Oil red O staining of livers from F0-CON dams [(a): 200X; (b): 400X] and F0-HFD dams [(c): 200X; (d): 400X] after full-term pregnancy. **(C)** Comparison of hepatic TG and TCHO levels between F0-CON and F0-HFD dams after full-term pregnancy. **(D)** Comparison of cytokines in umbilical cord blood between F0-CON and F0-HFD dams by ELISA. **(E)** Cytokine protein levels in placental tissues between F0-CON and F0-HFD dams. **(F)** Comparison of cytokine protein levels in placental tissues between F0-CON and F0-HFD dams. **P < 0.01; *P < 0.05.

We further investigated the underlying factors responsible for the increase in inflammation and abnormal lipid metabolism in the neonates. Consistent with the previous study (19), 8 weeks' HFD led to the increase of the mean body weight of female rats when compared to the control rats (*P* < 0.05) accompanied by glucose intolerance (Table 1) before mating. After full-term pregnancy, all maternal rats in the HFD group developed NASH with vacuolar and macro-vesicular steatosis and lobular inflammation with a distinct microanatomy in the livers (Figures 2A,B). The levels of total cholesterol (458.91 ± 16.37 vs 319.28 ± 9.13 μmol/L, *P* < 0.01) and TG levels (323.79 ± 14.18 vs 117.14 ± 5.01 μmol/L, *P* < 0.01) in HFD rats were significantly higher than those in control groups (Figure 2C). In addition, increased expression of inflammatory cytokines including IL6 and IL-1β was observed in umbilical cord blood from HFD maternal rats (Figure 2D). The levels of IL6, IL-1β, and TNF-α in the placenta from HFD rats were significantly higher than those from control rats (Figures 2E,F). These results indicate that HFD in maternal rats triggers a pro-inflammatory response in the placenta, which might be one of the strong susceptible factors to the occurrence of abnormal inflammation and lipid metabolism in the livers of the neonates.

**Table 1:** Biochemical parameters of F0 female models.

	**F0-CON**	**F0-HFD**	***P*-value**
*N*	6	6	
**Body weight (g)**			
3 weeks old	77.72 ± 1.55	78.00 ± 2.07	0.68
11 weeks old	215.00 ± 2.88	255.00 ± 10.4	0.021^*^
**IPGTTs (11 weeks old)**			
Glucose G_0_ (mmol/L)	2.88 ± 0.60	4.43 ± 0.2	0.022^*^
Glucose G_30_ (mmol/L)	15.03 ± 1.33	24.07 ± 0.33	0.0030^**^
Glucose G_60_ (mmol/L)	9.63 ± 0.94	14.8 ± 1.57	0.045^*^
Glucose G_120_ (mmol/L)	6.10 ± 0.91	9.03 ± 0.19	0.037^*^
AUC (11 weeks old)	37.02 ± 5.40	57.52 ± 4.30	0.015^*^

*The data were represented as the mean ± SEM*.

** *P < 0.01*,

* *P < 0.05 vs. the control group*.

### rIL6 Increases the Expression of *SCD1* With Abnormal Lipid Metabolism in HepG2 Cell Lines Upon *in vitro* Treatment

**Figure 3:**
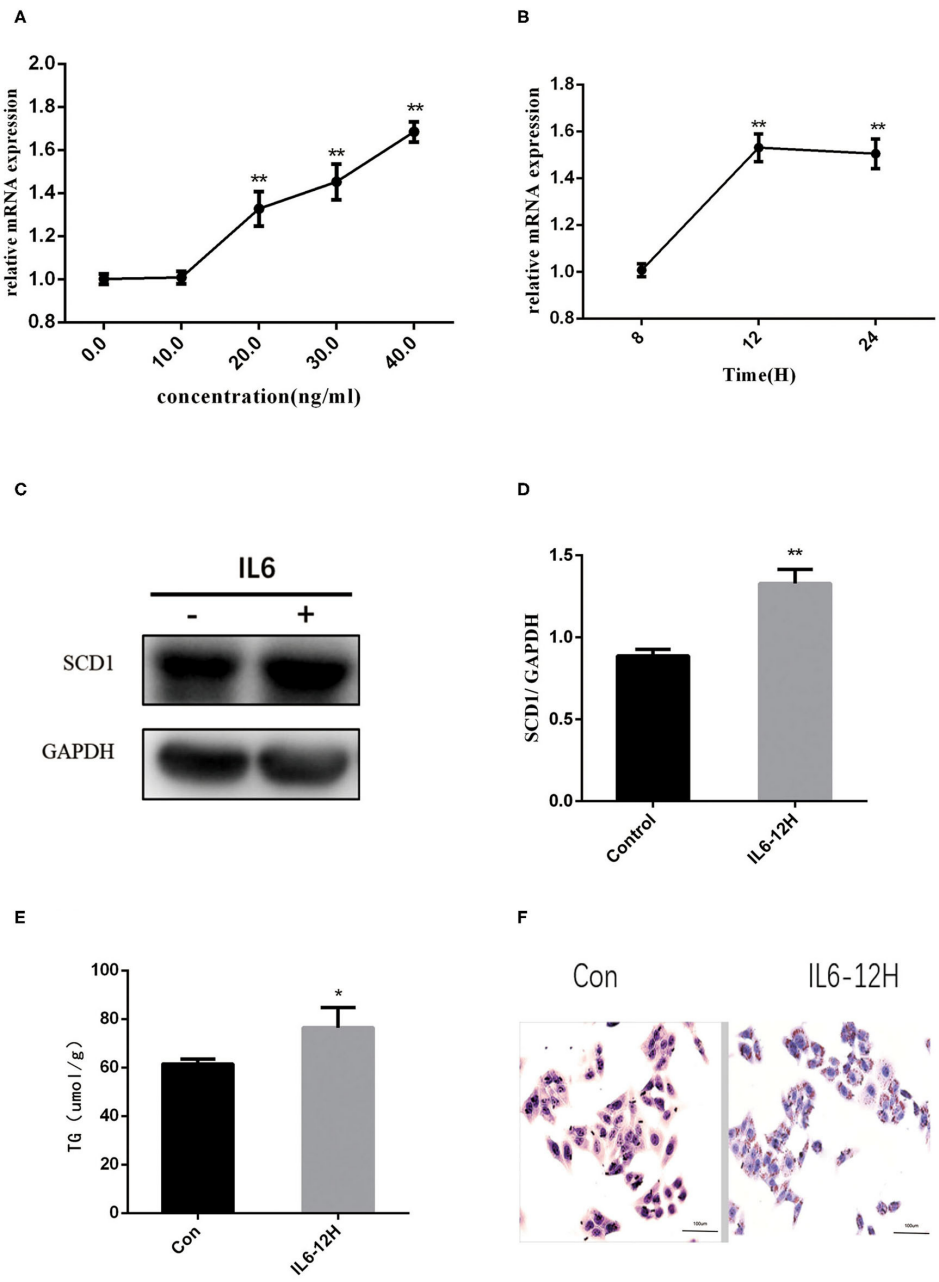
Recombinant interleukin-6 (rIL6) treatment induces an increase in SCD1 expression and lipid metabolism in HepG2 cells. **(A)** *Scd1* mRNA expression levels in HepG2 cells upon rIL6 treatment at different concentrations. **(B)** *Scd1* mRNA expression levels in HepG2 cells upon rIL6 treatment at different time points. **(C)** SCD1 protein levels in HepG2 cells with the treatment of rIL6 at 40 ng/μl for 12 h. **(D)** The relative protein levels of SCD1 in HepG2 cells with the treatment of rIL6 at 40 ng/μl for 12 h. **(E)** TG levels in HepG2 cells with the treatment of rIL6 at 40 ng/μl for 12 h. **(F)** Oil red O staining of HepG2 cells. a: Control group; b: HepG2 cells upon rIL6 treatment at 40 ng/μl for 12 h. **P < 0.01; *P < 0.05.

Considering the increase in the inflammatory cytokines in the placenta and abnormal lipid metabolism in the neonate livers, we further explored the mechanisms of how inflammatory cytokines regulate liver metabolism. As a higher expression of IL6 was observed in both intrauterine environment and neonatal livers, we used HepG2 cells to further explore the roles of IL6 in abnormal liver lipogenesis *in vitro*. Results from Q-PCR indicated that rIL6 treatment led to the increase in the expression of *SCD1* in time- and dose-dependent manners (Figures 3A,B). Treatment of 40 ng/μl rIL6 for 12 h led to an ~1.6-fold increase in *SCD1* mRNA expression in HepG2 cells when compared to the untreated cells. Protein level of SCD1 was also significantly elevated upon rIL6 treatment (Figures 3C,D). Moreover, intracellular TG content was augmented (76.47 ± 6.47 μmol/L) after rIL6 treatment when compared to untreated HepG2 cells (61.25 ± 1.25 μmol/L) (Figure 3E) with increased lipid droplets in rIL6 groups by oil red O staining (Figure 3F). These results indicate that IL6 might increase *SCD1* expression with abnormal lipid metabolism in HepG2 cells *in vitro*.

### Overexpression of *SCD1* Can Aggravate Intracellular Lipid Deposition

**Figure 4:**
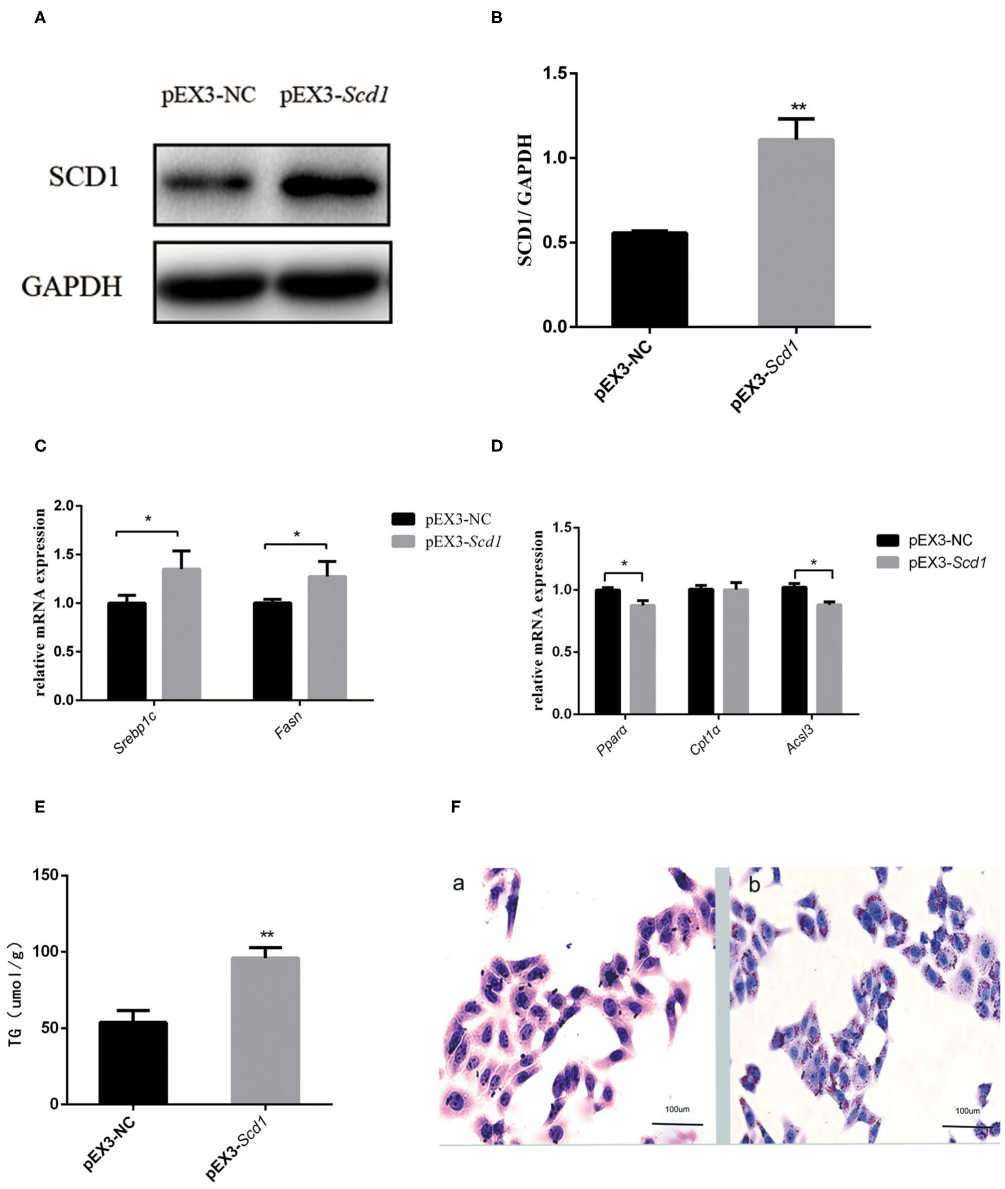
SCD1 overexpression promotes the intracellular lipid accumulation in HepG2 cells. HepG2 cells were transfected with pEX3-NC or pEX3-*Scd1* vectors. SCD1 protein levels were determined by western blot **(A)** and quantitatively compared **(B)** between two group cells. Meanwhile, mRNA levels of lipid synthesis-related genes **(C)**, lipid oxidation-related genes **(D)**, and intracellular TG levels **(E)** were compared as well between HepG2 cells transfected with pEX3-NC and pEX3-*Scd1* vectors. Oil red O staining was performed **(F)** to observe the lipid droplets in HepG2 cells transfected with either pEX3-NC (a) or pEX3-*Scd1* vectors (b). **P < 0.01; *P < 0.05.

In order to explore the direct association between SCD1 and lipid metabolism, we overexpressed *SCD1* in HepG2 cells through transfection (Figures 4A,B). We found that lipid synthesis-related genes such as *SREBP1c* and *FASN* were upregulated (Figure 4C), whereas β-oxidation-related genes such as *PPAR*α and *ACSL3* were downregulated when compared with control cells (Figure 4D). Consistently, the content of intracellular TG was increased to 95.87 ± 4.05 μmol/L with the overexpression of *SCD1* in HepG2 cells, which was significantly higher than that from vector control cells (53.71 ± 3.71 μmol/L) (Figure 4E). The results of oil red staining also showed more lipid droplets, which were similar to TG content (Figure 4F).

### *SCD1* Knockdown Reduces Lipid Accumulation in HepG2 Cells Under Inflammatory Conditions

**Figure 5:**
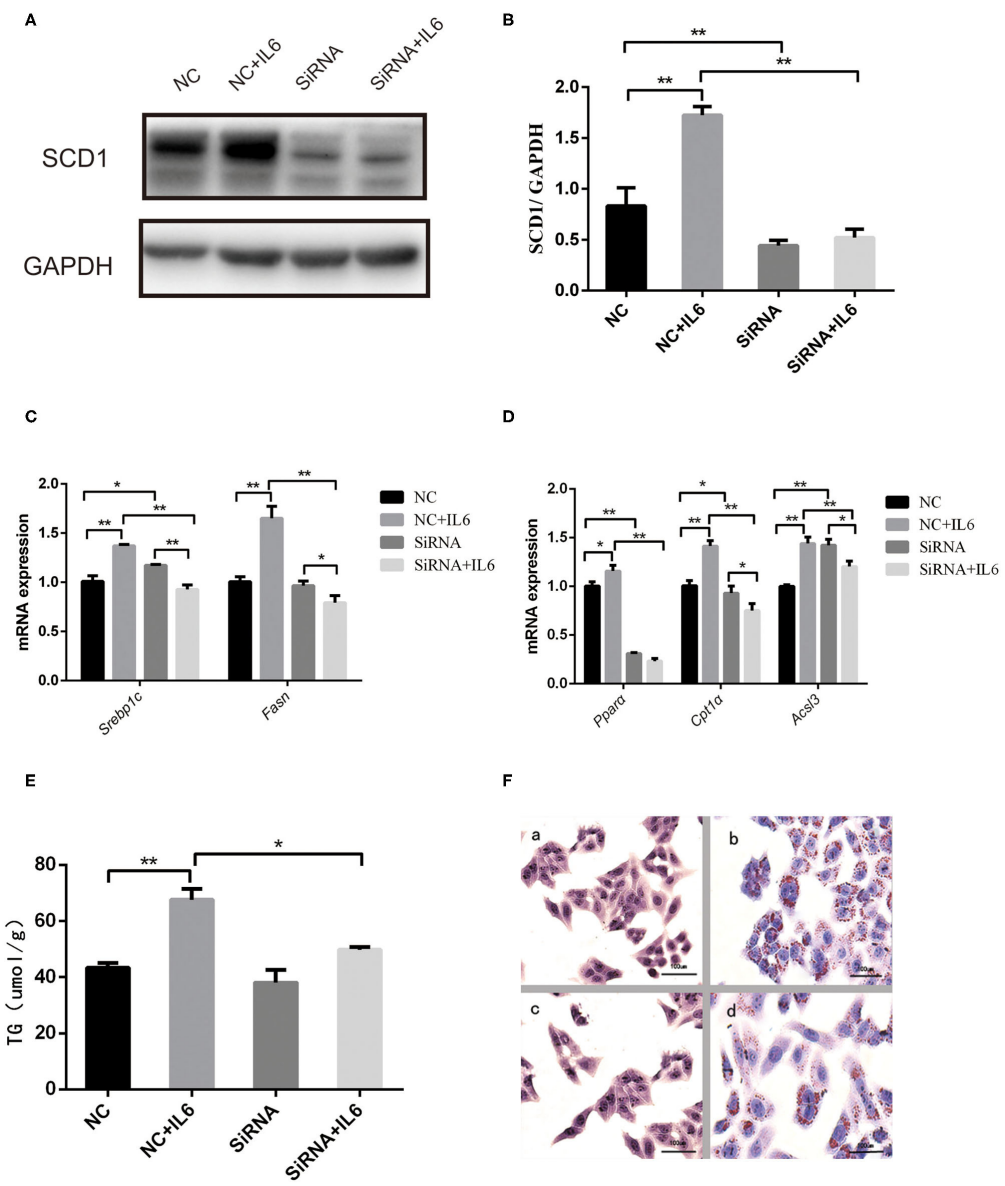
*Scd1* knockdown alleviates the lipid accumulation in HepG2 cells exposed to rIL6. **(A)** Determination of SCD1 protein levels upon siRNA interference in HepG2 cells with or without rIL6 treatment *in vitro*. **(B)** Quantitative comparison of SCD1 protein levels in HepG2 cells upon siRNA knockdown with or without rIL6 treatment *in vitro*. **(C–E)** Quantitative comparison of mRNA levels of genes related to lipid synthesis **(C)** and lipid oxidation **(D)** and intracellular TG levels **(E)** in HepG2 cells upon siRNA knockdown with or without rIL6 treatment *in vitro*. **(F)** Oil red O staining of HepG2 cells. (a): Negative control (NC) group; (b): NC+IL6 group; (c): siRNA group; (d): siRNA+IL6 group. **P < 0.01; *P < 0.05.

Interference of SCD1 expression by siRNA was performed to further validate the role of SCD1 in the regulation of lipid metabolism (Figures 5A,B). However, knockdown of *SCD1* did not decrease intracellular TG content when compared to the NC group (Figure 5E). Since rIL6 treatment induced the overexpression of both lipid synthesis-related genes and β-oxidation-related genes in HepG2 cells (Figure 5D), we further analyzed the effects of *SCD1* knockdown upon rIL6 treatment. We found that when *SCD1* was knocked down in HepG2 cells, the expressions of lipid synthesis-related genes (*SREBP1c* and *FASN*) (Figure 5C) and oxidation-related genes (*PPAR*α, *CPT1*α, and *ACSL3*) were downregulated upon rIL6 treatment when compared to untreated cells (Figure 5D). TG content was decreased from 67.63 ± 3.87 to 49.79 ± 0.96 μmol/L (Figure 5E). Lipid droplets decreased upon rIL6 treatment as well as in Scd1-knockdown HepG2 cells (Figure 5F). These results suggested that *SCD1* might be one of the key negative regulators of lipid metabolism in HepG2 cells under a proinflammatory condition.

### Upregulation of *Scd1* Increases TG Synthesis in the Liver of Neonatal Rats

**Figure 6:**
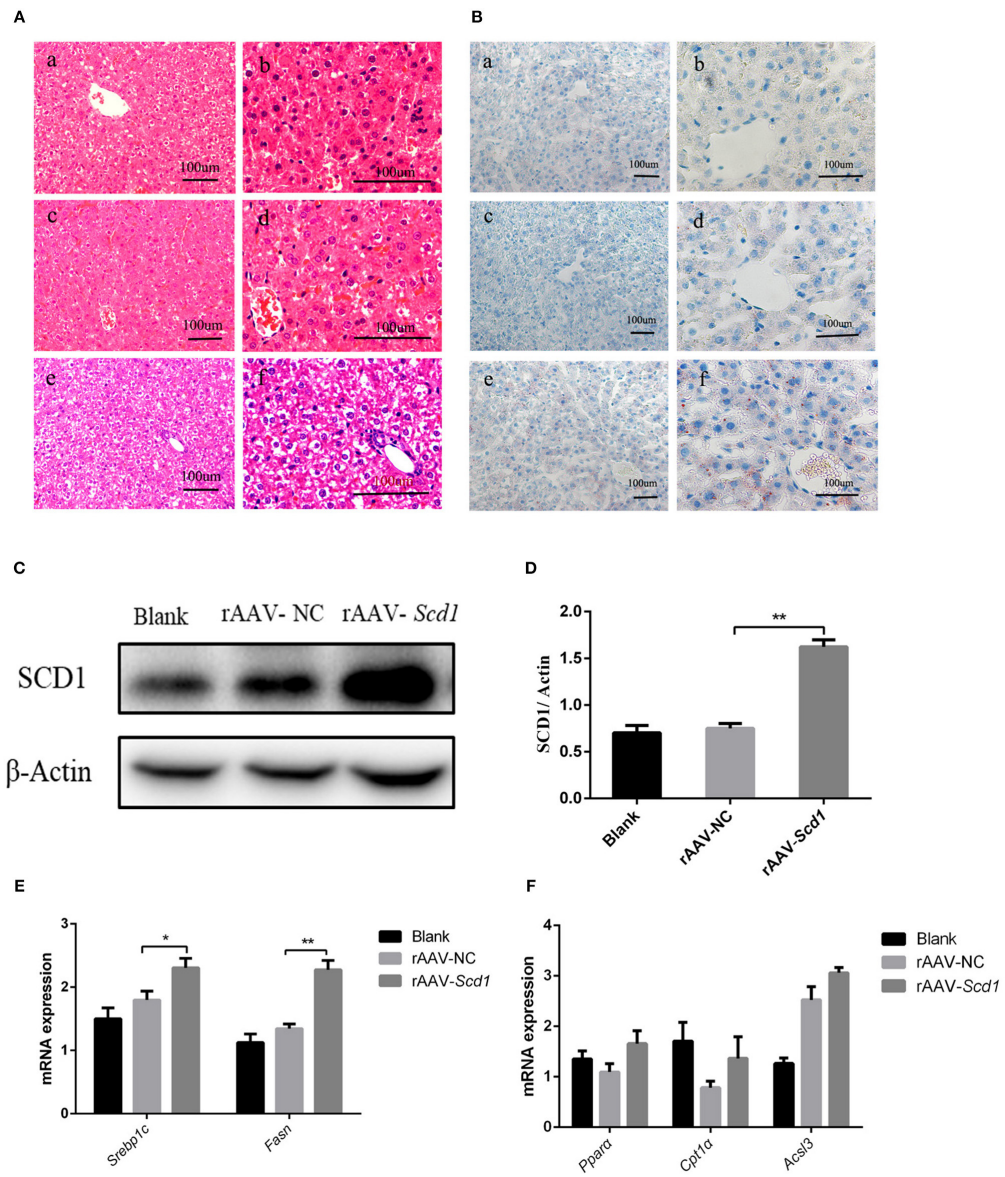
Liver pathology and gene expression in the rats at 3 weeks old after rAAV injection after birth. H&E staining **(A)** and oil red O staining **(B)** were performed in the livers from the rats of blank [(a): 200X; (b): 400X], rAAV-NC-injected [(c): 200X; (d): 400X], and rAAV-*Scd1*-injected groups [(e): 200X; (f): 400X] at 3 weeks old. SCD1 protein levels in livers of 3-week-old rats were detected by western blot **(C)** and compared among blank, rAAV-NC-injected, and rAAV-*Scd1*-injected groups. Quantitative comparison of *Scd1* protein levels in livers of 3-week-old rats were also compared **(D)**. Comparison of mRNA levels of genes related to lipid synthesis **(E)** and lipid oxidation **(F)** in the livers of 3-week-old rats was determined in blank, rAAV-NC, and rAAV-*Scd1* groups at the same time. **P < 0.01; *P < 0.05.

To investigate the role of *Scd1* in regulating liver lipid metabolism *in vivo*, rAAV-Scd1-overexpressing vectors and rAAV-NC vectors were injected in tail veins in neonatal rats. After weaning, the body weight and glucose tolerance showed no significant difference between vector-injected F1 neonatal and blank rats (*P* > 0.05; Table 2). Liver lipid metabolism-related genes and lipid content were detected in three F1 offspring groups. Our results indicated that TG content in the livers from rAAV-Scd1-treated F1 neonatal rats (1,691.78 ± 70.68 μmol/L) was much higher than that from rAAV-NC-treated F1 neonatal rats (1,324.20 ± 109.75 μmol/L) (*P* < 0.05). However, there was no significant difference in cholesterol content among three F1 neonate groups (Table 2). Histopathological analysis also showed that the liver cells of F1 offspring rats at 3 weeks of age exhibited a balloon-like and swollen shape with more extent with the injection of the rAAV-Scd1 vector (Figure 6A). More red-lipid droplets were visible around the nucleus of the hepatocytes of F1-rAAV-Scd1 rats (Figure 6B) with the upregulation of *Scd1* expression levels (Figures 6C,D). The expression levels of fatty acid synthesis-related genes (*SREBP1c* and *FASN*) in the livers of the F1-rAAV-Scd1 group were significantly upregulated compared with those of the F1-rAAV-NC group (Figure 6E; *P* < 0.05). Although the expression levels of β-oxidation-related genes including *Ppar*α, *Cpt1*α, and *Acsl3* were slightly increased, no significant difference was found (Figure 6F). Taken together, it could be hypothesized that the upregulation of *Scd1* from the birth might trigger abnormal lipid metabolism in the livers of the offsprings even with normal diet during the pregnancy.

**Table 2:** Biochemical parameters of newborn rats injected with rAAV.

	**Blank**	**rAAV-NC**	**rAAV-*Scd1***	***P*-value**
*N*	7	7	7	
**Body weight (g)**				
Day 1	6.43 ± 0.16	6.26 ± 0.30	6.46 ± 0.25	0.57
Day 7	17.40 ± 0.55	20.44 ± 0.61	20.64 ± 0.45	0.77
Day 14	37.16 ± 0.83	40.54 ± 0.95	41.2 ± 0.64	0.58
Day 21	69.10 ± 1.53	76.39 ± 1.41	76.12 ± 1.47	0.90
**IPGTTs (3 weeks old)**				
Glucose G_0_ (mmol/L)	3.50 ± 0.18	3.24 ± 0.23	3.69 ± 0.19	0.14
Glucose G_30_ (mmol/L)	13.48 ± 0.97	12.50 ± 1.04	14.01 ± 1.01	0.30
Glucose G_60_ (mmol/L)	5.02 ± 0.61	7.09 ± 0.57	5.84 ± 0.53	0.14
Glucose G_120_ (mmol/L)	3.36 ± 0.57	3.90 ± 0.74	3.07 ± 0.38	0.38
AUC (3 weeks old)	26.12 ± 1.90	28.67 ± 1.58	27.69 ± 0.91	0.97
TG (μmol/L)	1,289.3 ± 104.6	1,324.2 ± 109.8	1,691.8 ± 70.7	0.021^*^
TCHO (μmol/L)	357.8 ± 36.5	413.6 ± 10.8	406.7 ± 36.7	0.88

*The data were represented as mean ± SEM*.

* *P < 0.05 rAAV-NC group vs. rAAV-Scd1*.

## Discussion

We found that maternal HFD during pregnancy resulted in weight gain, impaired glucose tolerance, and the occurrence of NAFLD in the offsprings at 3 weeks of weaning in a previous study (19). Here, we traced back to define how maternal HFD affects lipid metabolism as well as its regulation by inflammatory pathways in neonatal liver. Our results suggested that maternal nutritional status during pregnancy could be among the key drivers of pathogenesis of NAFLD in the neonates. Maternal HFD could upregulate hepatic *Scd1* expression in F1 neonates and led to NAFLD in the livers of F1 neonates. This could be mediated by an upregulation of inflammatory cytokines such as IL6 at the maternal–fetal interface. Our findings thus provided new clues on how maternal diet affects lipid metabolism and inflammation in the neonates.

Current evidence supports that inappropriate maternal nutrition leads to liver metabolic disorders, mitochondrial dysfunction (7, 24), intestinal flora imbalance (25), and intrahepatic macrophage activation (26), ultimately making the offsprings more prone to NAFLD after birth. However, the underlying mechanisms remain unclear. In this study, we have found that maternal HFD induced intrauterine inflammation, which was demonstrated by the increase in inflammatory cytokines (IL6, IL-1β, and TNF-α) in umbilical cord blood and the placenta. It is already well addressed that IL6, IL-1β, and TNF-α were closely related to the occurrence of NAFLD (27), which led to the production of acute reaction proteins and subsequently cause a series of inflammatory reactions in the liver. Upregulation of proinflammatory cytokines including IL6, IL-1β, and TNF-α were observed in placental tissue, while higher levels of IL6 and IL-1β were present in umbilical cord blood in HFD pregnant rats. The spectrum of inflammatory cytokines in the placenta and umbilical cord blood is quite similar except for the increase of TNF-α in the placenta. The inflammatory factors in the placenta represent the maternal condition. Monocyte infiltration in HFD-fed maternal circulation might be responsible for the release of IL6 and TNF-α in the fetus (28). Previous study showed that the expression level of TNF-α in HFD dams from normal-diet maternity was significantly higher than that of normal-diet dams from HFD maternity reference (29). Therefore, we speculated that the level of TNF-α in the process of NAFLD was more affected by the diet of neonatal rats. It was reported that maternal HFD triggered the inflammation in the fetal circulation, liver, and brain (16). Our results demonstrated that the increase of inflammatory cytokines in F1-HFD generation was correlated with cytokine levels in umbilical cord blood. Interestingly, we found that both IL6 and IL-1β were elevated in the placenta, umbilical cord blood, and fetal hepatic tissue. It is well-recognized that IL6 is an important inflammatory cytokine in the development of fatty liver through the activation of the GP130-STAT3 axis (30). The expression of IL6 was observed in hepatic cells of two F1 neonate groups, suggesting that IL6 might be a key mediator of maternal intrauterine inflammation and the onset of fetal NAFLD.

The relationships between inflammation and NAFLD-related metabolic disorders have been intensively discussed (31). The occurrence of inflammation is the orchestration of genetics, diet, behavior, and microbiome factors. Our results further indicated that intrauterine inflammatory environment emerged as a potential contributor aggravating the metabolic disorders in F1 neonates especially that with HFD during pregnancy. To explore the exact mechanisms triggered by IL6, we performed an *in vitro* assay using HepG2 cell lines. We found that rIL6 treatment significantly increased lipid droplets and TG content. Notably, the expression of *SCD1* was upregulated in HepG2 cells. Considering that *SCD1* is expressed at high levels in lipogenic tissues such as liver and white adipose, we thus proposed that *SCD1* might be one of the mediators affected by intrauterine IL6 in the liver of F0-HFD neonates.

A fetus relies mainly on amino acids and carbohydrates (glucose and lactate) for oxidative metabolism. Once the expression of genes involved in fatty acid metabolism is inhibited, the metabolism gradually switches to lipid-based fuels after birth (32). Our results showed that both lipid synthesis- and β-oxidation-related genes were upregulated in F0-HFD neonate offsprings. Maternal HFD affects the liver TG content of F0-HFD offsprings as well. Combined with the *in vitro* assay, it is clear that *Scd1* plays a key role in abnormal lipid metabolism in the neonates from F0-HFD maternity.

SCD1 catalyzes the rate-limiting step for the conversion of SFA into MUFAs. It also plays a critical role in the incidence of NAFLD under HFD. Alteration of *SCD1* expression was demonstrated to affect lipid metabolism genes. For instance, hepatic *Scd1* deficiency impaired the transcription of lipogenic genes including *Srebp1c, Fas*, and *Acc* and improved hepatic steatosis caused by a high-carbohydrate diet (33). Downregulation of *Scd1* attenuates leptin-induced phosphorylation of signal transducers and activator of transcription 3 (STAT3), leading to obesity (34). In our study, *SCD1* overexpression in HepG2 cells significantly increased lipid synthesis-related genes and decreased β-oxidation-related genes. Knockdown of *SCD1*, on the contrary, reversed the increase of intracellular TG and inhibited the expression of lipid synthesis-related genes caused by rIL6 treatment. These results indicated that *SCD1* played an important role in lipid synthesis through the regulation of lipid synthesis gene expression. Results from overexpression of *Scd1* in neonatal rats were consistent with the *in vitro* results. Previous studies revealed that *SCD1* increased the expression of lipid synthesis genes by the upregulation of *Lxr* genes (35) and the activation of the *Ampk-Srebp1c* axis (36, 37). *Srebp1* is an important transcription factor of the lipid metabolism pathway. In addition, Liu et al. reported that the expression of *SCD1* could be regulated by microRNA, such as miR-125b, miR-29a, and miR-192-5p (38). Although the underlying mechanism needs to be further investigated, the current study indicated that *SCD1* played key roles in fetal NAFLD under maternal HFD.

Since *SCD1* is mainly expressed in lipogenic tissues such as liver and adipose tissues (39), the possibility to consider SCD1 as a potential therapeutic target for the treatment of metabolic disorders including obesity and NASH has been investigated in previous studies (40). Liver-specific *Scd1*-knockout mice were resistant to obesity and hepatic steatosis caused by a high-carbohydrate diet, with no side effects (33). Global *Scd1* depletion increases insulin sensitivity and glucose utilization (41). Besides, *Scd1*-deficient mice exhibited resistance to metabolic stress and possessed augmentation in beige adipocytes under basal conditions by regulating the differentiation of preadipocytes from white adipogenesis to beige adipogenesis, indicating that *SCD1* shows promise for counterbalancing obesity and metabolic diseases (42). Exogenous infusion of *SCD1* antisense oligonucleotide (ASO), which is mostly distributed in the liver, can protect mice from weight gain and insulin resistance caused by HFD without any mechanism-based side effects (43). Our results gave a clue for the mechanism by which the maternal HFD leads to the occurrence of NAFLD in the offsprings. However, inhibition of *SCD1* activity in pancreatic β-cell induces apoptosis of β cells, changes the synthesis of intracellular membrane phospholipids, and destroys the fusion of autophagosome and lysosome (44). Furthermore, although *Scd1* deficiency protected mice from liver steatosis, these mice displayed severe hepatic damage, which is promptly rescued by oleic acid endogenous supplementation (45). The contribution of *Scd1* to NAFLD is still not fully depicted, but our results provided some clues for the treatment of NAFLD in adolescents. In addition, the current results suggested that both IL6 and IL-1β were elevated in the placenta, umbilical cord blood, and fetal hepatic tissue. In this study, we explored only the role of IL6, so the roles of other inflammatory factors in the development of NAFLD need to be explored in future studies. Furthermore, it is known that maternal HFD programs offspring liver steatosis in a sexually dimorphic manner (46). The male-to-female ratio in this study is 1:1 in each group. Further study is needed to investigate offspring liver steatosis in a sexually dimorphic manner. In addition, there has been a growing interest regarding the association between NAFLD and the type of HFD; previous studies showed that moderate intake of unsaturated fatty acids had a protective effect on the development of NAFLD and that SFAs seemed to be prone to hepatic steatosis (47). Therefore, as the rate-limiting enzyme that catalyzes the rate-limiting step for the conversion of SFA into MUFA, inhibition of *SCD1* activity might ameliorate the metabolic disorders with less adverse effects caused by HFD in the future (48).

## Conclusions

In summary, our results provided direct evidence of the effects of maternal HFD on the occurrence of NAFLD in offsprings at a very early time point of birth. Upregulation of hepatic *Scd1* expression in F1 neonates was one of the key factors triggered by intrauterine inflammatory cytokine. Anti-scd1 and anti-inflammatory therapy could be options for management of obesity and metabolic disorders in future clinical practice. However, it should be noticed that the modulation/improvisation of maternal diet/nutrition toward a healthier balanced diet accompanied by appropriate physical activity and maintenance of optimum weight during pregnancy was the most important way to prevent the occurrence of NAFLD in offsprings.

## Data Availability Statement

The datasets presented in this study can be found in online repositories. The names of the repository/repositories and accession number(s) can be found in the article/Supplementary Material.

## Ethics Statement

The animal study was reviewed and approved by the University Animal Use Committee.

## Author Contributions

BC, CL, and YD conceived and designed the study. BC and QZ performed the experiments. BC analyzed the data and wrote the paper. All authors read and approved the final manuscript.

## Conflict of Interest

The authors declare that the research was conducted in the absence of any commercial or financial relationships that could be construed as a potential conflict of interest.

## Abbreviations

NAFLD: nonalcoholic fatty liver disease
SCD1: stearoyl-coenzyme A desaturase 1
PPARα: peroxisome proliferator activated receptor α
SREBP1c: sterol regulatory element-binding transcription factor 1
CPT1α: carnitine palmitoyl transferase 1α
ACSL3: acyl-CoA synthetase long-chain family member 3
FASN: fatty acid synthase
IL6: interleukin-6
IL-1β: interleukin-1β
TNF-α: tumor necrosis factor-α
TG: triglyceride
CHO: cholesterol.
