# Supplementary material for: Maternal High-Fat Diet Leads to Non-alcoholic Fatty Liver Disease Through Upregulating Hepatic SCD1 Expression in Neonate Rats
Source: Front Nutr. 2020 Nov 17;7:581723. doi: 10.3389/fnut.2020.581723 (PMC7705221; doi:10.3389/fnut.2020.581723)
Supplement: Supplementary file 1 [file Table_1.DOCX]

Supplementary Material

**Supplementary Tables:**

Table S1. Q-PCR primer sequences used for mRNA quantification from SD rats.

| Gene | Primer sequence |
| --- | --- |
| IL6 | Forward: 5ˊ-ACTGGTCTGTTGTGGGTGGT-3ˊ |
|  | Reverse: 5ˊ-AGTTGCCTTCTTGGGACTGA-3ˊ |
| IL-1β | Forward: 5ˊ-GCTGTGGCAGCTACCTATGTCTTG-3ˊ |
|  | Reverse: 5ˊ-AGGTCGTCATCATCCCACGAG-3ˊ |
| TNF-α | Forward: 5ˊ-AAATGGGCTCCCTCTCATCAGTCC-3ˊ |
|  | Reverse: 5ˊ-TCTGCTTGGTGGTTTGCTACGAC-3ˊ |
| Srebp1c | Forward: 5ˊ-GCGCTACCGTTCCTCTATCA-3ˊ |
|  | Reverse: 5ˊ-GGATGTAGTCGATGGCCTTG-3ˊ |
| Fasn | Forward: 5ˊ-GGAACTGCTTTCTCTTTCTGC-3ˊ |
|  | Reverse: 5ˊ-AACGCTCCTCTTCAACTCCA-3ˊ |
| Scd1 | Forward: 5ˊ-TGTTCGTCAGCACCTTCTTG-3ˊ |
|  | Reverse: 5ˊ-AGTTGATGTGCCAGCGGTA-3ˊ |
| Pparα | Forward: 5ˊ-CCATACAGGAGAGCAGGGATT-3ˊ |
|  | Reverse: 5ˊ-CCACCATTTCAGTAGCAGGA-3ˊ |
| Cpt1α | Forward: 5ˊ-ATCCACCATTCCACTCTGCT-3ˊ |
|  | Reverse: 5ˊ-TGTGCCTGCTGTCCTTGATA-3ˊ |
| Acsl3 | Forward: 5ˊ-TAACGGAACTTGGGAAGAGC-3ˊ |
|  | Reverse: 5ˊ-AAGGCATCAGTCACCAGACC-3ˊ |
| 18S | Forward: 5ˊ-AAGTTTCAGCACATCCTGCGAGTA-3ˊ |
|  | Reverse: 5ˊ-TTGGTGAGGTCAATGTCTGCTTTC-3ˊ |

Table S2. Q-PCR primer sequences used for mRNA quantification in HepG2 cells.

| Gene | Primer sequence |
| --- | --- |
| Srebp1c | Forward: 5’-GCTGTTGGTGCTCGTCTCCTTG-3’ |
|  | Reverse: 5’-GCTTGCGATGCCTCCAGAAGTAC-3’ |
| Fasn | Forward: 5’- CCTGGCTGCCTACTACATCG-3’ |
|  | Reverse: 5’- CACATTTCAAAGGCCACGCA-3’ |
| Scd1 | Forward: 5’-GCGATATGCTGTGGTGCTTAATGC-3’ |
|  | Reverse: 5’-GGAGTGGTGGTAGTTGTGGAAGC-3’ |
| Pparα | Forward: 5’-TCCTCGGTGACTTATCCTGTGGTC-3’ |
|  | Reverse: 5’-GCGTGGACTCCGTAATGATAGCC-3’ |
| Cpt1α | Forward: 5’-CAGACACCATCCAGCACATGAGAG-3’ |
|  | Reverse: 5’-TGAGGCTCCGAGGTATTGTCCAG-3’ |
| Acsl3 | Forward: 5’- ACCAACATCGCCATCTTCTGTGAG-3’ |
|  | Reverse: 5’-TGCATGAACAATGGCTGGACCTC-3’ |
| 18S | Forward: 5’-AAGTTTCAGCACATCCTGCGAGTA-3’ |
|  | Reverse: 5’-TTGGTG AGGTCAATGTCTGCTTTC-3’ |

**
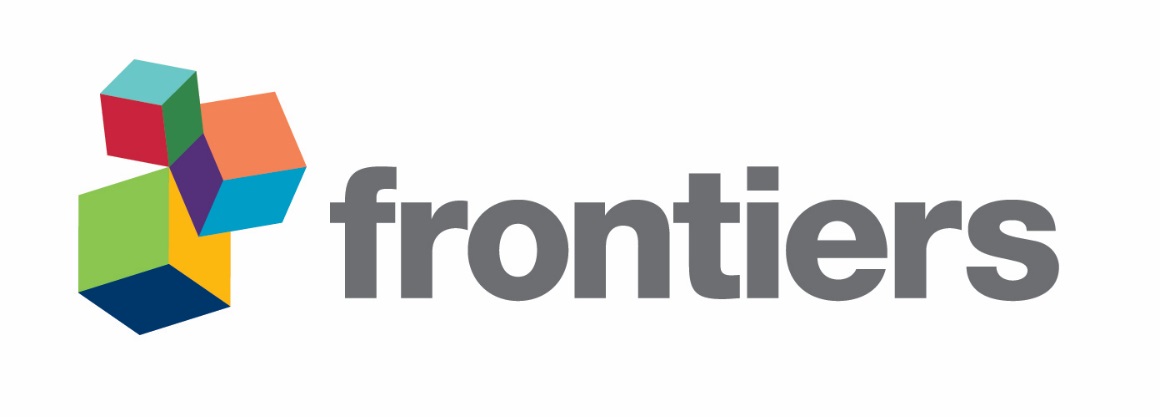
**
